# A comprehensive coin dataset highlighting the numismatic heritage of Bangladesh

**DOI:** 10.1016/j.dib.2025.112212

**Published:** 2025-10-30

**Authors:** Mahamudul Hasan, Krittika Roy, Nowshin Tasnia, Mohammad Rifat Ahmmad Rashid

**Affiliations:** Department of Computer Science and Engineering, East West University, Aftabnagar, Dhaka, Bangladesh

**Keywords:** Bangladeshi coins, Denomination, Classification, Coin verification, Banking transactions

## Abstract

Following Bangladesh's independence in 1971, the country introduced Taka coins in 1973, featuring a lotus flower and denomination, symbolizing its rich history and cultural heritage. In 2013, a new coin series was issued, displaying the portrait of Bangabandhu Sheikh Mujibur Rahman on the obverse and the national emblem on the reverse. This series included denominations of 1, 2, and 5 Taka coins. In this research, we present a dataset of 9983 images representing 26 different classes of Bangladeshi coins. The dataset includes coins of various denominations, such as 1, 5, 10, 25, and 50 Poisha, as well as 1, 2, and 5 Taka coins. Each image captures the coins from both the obverse and reverse sides, showcasing different shapes, sizes, and designs under diverse environmental conditions and backgrounds. The primary significance of this dataset lies in its ability to assist visually impaired individuals in daily banking transactions. By addressing challenges related to coin detection, recognition, and the integrity of monetary systems, this dataset contributes to the fields of computer vision, artificial intelligence, and machine learning. The technology empowered by this dataset can enable visually impaired individuals to independently and accurately identify and differentiate between various denominations, thus enhancing their participation in the financial sector.

Specifications Table

**Table:** 

Subject	The Numismatic Heritage of Bangladesh
Specific subject area	Image processing
Type of data	Raw Images Dataset
Data collection	The photographs for this research were captured using the Realme 9 5 G smartphone, which is equipped with a 48MP AI Triple Camera system. The primary camera features a 48MP Ultra High Definition (UHD) lens with an aperture of F1.8, providing a field of view (FOV) of 79.8° and an equivalent focal length of 25.4 mm. Additionally, the system incorporates a 6P lens with a sensor size of 1/2″. The camera setup also includes a 4 cm macro lens with 2 million pixels, an aperture of F2.4, a FOV of 88.8°, and a focus distance of 4 cm, as well as a portrait lens with 2 million pixels, an aperture of F2.4, a FOV of 88.8°, and an equivalent focal length of 21.8 mm using a 3P lens. This paper presents a dataset of 9983 images of Bangladeshi coins, spanning 26 different classes. The dataset provides critical support for coin recognition and detection, especially aiding visually impaired individuals in performing financial transactions independently.
Data source location	East West University A/2, Jahurul Islam Avenue Jahurul Islam City, Aftabnagar Dhaka-1212, Bangladesh Latitude and longitude: 23.7736° N, 90.4227° E
Data accessibility	Repository name: Mendeley Data Data identification number: 10.17632/w5zspm82zd.1 Direct URL to data: https://data.mendeley.com/datasets/w5zspm82zd/1

## 1 Value of the data

• This dataset contains 9983 images of Bangladeshi coins across 26 classes, covering both obverse and reverse sides under varied lighting and backgrounds, and is openly available in Mendeley Data.
• It can benefit researchers in computer vision, machine learning, accessibility technology, and also numismatists and collectors.
• The data can be reused for benchmarking classification, detection, and segmentation models, and for testing automatic or interactive annotation workflows under real-world variability.
• The inclusion of both phased-out Poisha and currently circulating Taka denominations, photographed with detailed consistency, makes the dataset particularly valuable for historical, technological, and accessibility-focused applications.
• Supports assistive technologies for denomination recognition, counterfeit screening and sorting in operational settings, and digital archiving for scholars and collectors.

## 2 Background

This dataset of 9983 images, encompassing 26 different classes of Bangladeshi coins including 1 Poisha, 5 Poisha, 10 Poisha, 25 Poisha, 50 Poisha, 1 Taka, 2 Taka, and 5 Taka, contributes significantly to artificial intelligence, computer vision, and machine learning by addressing challenges related to coin recognition, classification, and currency integrity. By capturing coins of various shapes and sizes, photographed from both sides under diverse conditions, the dataset enables the development of advanced recognition models that can assist visually impaired individuals in confidently managing daily financial transactions. In parallel with such dataset-driven efforts, recent advancements in object-based annotation and segmentation workflows have further transformed the field of image-based recognition. Cutting-edge frameworks now leverage deep learning to automatically annotate class-specific object instances under diverse imaging conditions, greatly reducing the manual effort needed for dataset curation. For instance, transformer-enhanced detectors and hybrid architectures have significantly improved instance-level classification performance across complex scenes [1,2].

In addition to its role in accessibility and computer vision, this dataset also holds significant value for coin collectors and numismatists. Coin collecting is not only a popular hobby but also an important means of preserving cultural and economic history. By documenting both circulated and phased-out denominations, the dataset provides collectors with a structured digital archive that can assist in cataloging and classifying collections, verifying authenticity through comparison with reference images, identifying mint variations and anomalies, and tracing historical changes in iconography, material, and denomination. Thus, even as the use of physical currency declines in the digital era, this dataset continues to support numismatic research and the collecting community by safeguarding the visual and historical legacy of Bangladeshi coinage.

## 3 Data description

Following the partition of Bengal in 1947, East Bengal became the eastern region of Pakistan, later renamed East Pakistan in 1956. During this period, the term "taka" was used on official notes and coins alongside the Pakistani rupee. Bangladesh continued using the Pakistani rupee, which was decimalized in 1961 into 100 Poisha per rupee. In 1972, the Bangladeshi taka replaced the Pakistani rupee at par, with subdivisions into 100 Poisha. Coins as small as 1 Poisha were produced, although today the lowest denomination typically in circulation is the 1 taka coin.

In 1973, coins with values of 5, 10, 25, and 50 Poisha were introduced, followed by the 1 taka coin in 1975. These coins were made from aluminum, steel, or copper-nickel. Over time, higher denominations were introduced, including the 1 taka coin in 1994 and the steel 2 taka coin in 2004 and 2008. Due to inflation, however, coins of lower denominations, such as Poisha, are no longer widely circulated. While 1, 2, and 5 taka coins remain in occasional circulation, the most recent coins were issued in 2013. Coins are not released annually, and the Bangladesh Bank is responsible for issuing currencies of 10 taka and higher, while the Ministry of Finance oversees the release of 2 and 5 taka coins. The lower-denomination coins, including all Poisha coins, have been practically phased out due to inflation.

In addition to the comprehensive coverage of Bangladeshi coins described above, it is useful to contextualize this dataset within the broader landscape of currency image datasets. For example, Suryawanshi et al. [3] introduced an Indian coin image dataset focusing on prevalent circulating denominations, whereas Le Texier et al. [4] presented data on Euro coin distributions to study cross-border currency movement rather than enabling complex classification tasks. Meanwhile, note-oriented datasets, such as those for Kazakhstani banknotes [5] and Ghanaian currency [6], address different challenges and features inherent to paper money. Compared to these datasets, our collection offers a unique amalgam of historical and contemporary Bangladeshi coins, capturing both phased-out Poisha and currently circulated Taka denominations. Additionally, our dataset includes images taken under diverse environmental conditions and from multiple angles, facilitating more robust recognition performance. By encompassing both practical (e.g., assisting visually impaired users) and scholarly applications (e.g., numismatic research), our dataset fills an important niche, complementing and extending the scope of existing resources in the domain of currency recognition and analysis.

**Fig. 1:**
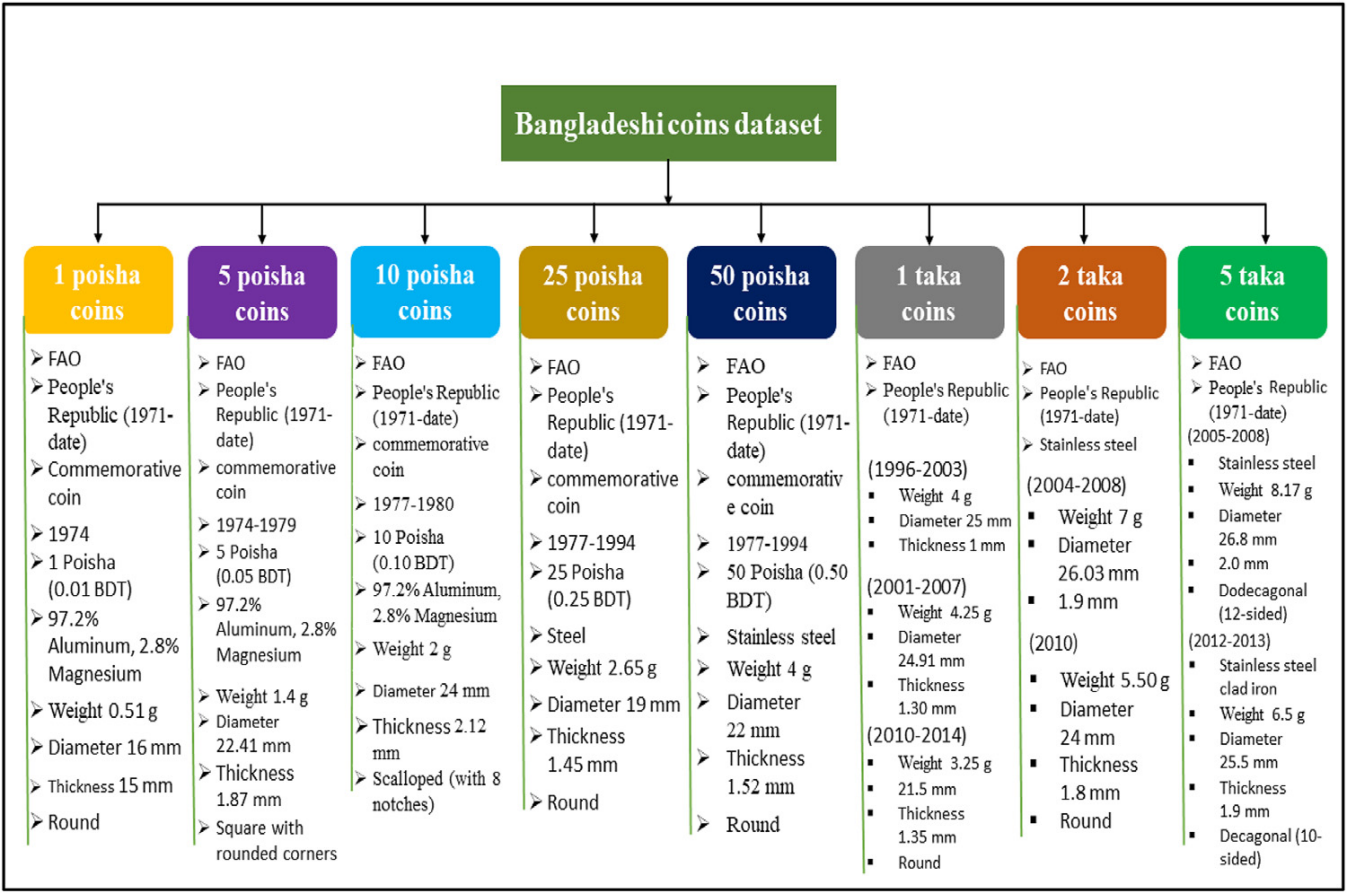
Features of Bangladeshi coins dataset.

This dataset addresses the gaps in previous collections by including all modern-day Bangladeshi coins, providing a comprehensive set of images. Fig. 1 illustrates the features of Bangladeshi coins dataset

The denominations included in the Bangladeshi coin dataset range from 1 Poisha to 5 Taka, covering 1 Poisha, 5 Poisha, 10 Poisha, 25 Poisha, 50 Poisha, 1 Taka, 2 Taka, and 5 Taka. These coins are made from various materials, including clad iron, steel, stainless steel, magnesium, and aluminum. The coins differ in thickness, diameter, and weight. The first 1 Poisha coin was introduced in 1974, while the 5 Taka coin was issued in 2010. The 10 Poisha coin, initially issued in 1977, was reissued in 1994. The Bangladeshi taka, which became the official currency after Bangladesh’s separation from Pakistan in 1971, serves as the country’s primary unit of currency. Table 1 presents several images from the dataset. As shown, the images display both the obverse and reverse sides of the coins under a variety of backgrounds, lighting conditions, and camera angles.

**Table 1:** Coin denomination.

### 3.1 One poisha coin

**Fig. 2:**
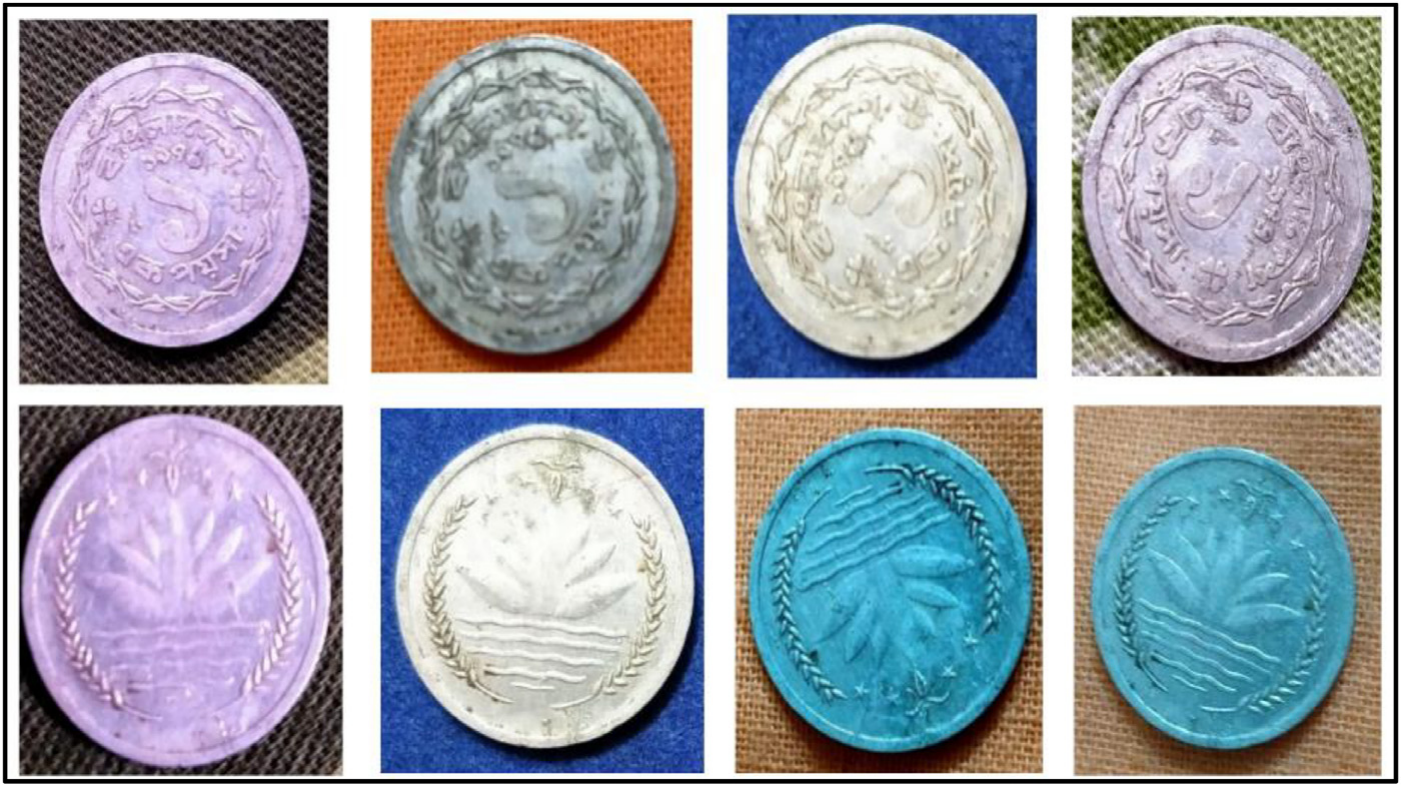
Images of 1 Poisha coin by the years.

As shown in Fig. 2, the front side of the 1 Poisha coin features the Bangladeshi national emblem, which includes four stars, rice sheaves, tea leaves, and a water lily. These elements symbolize the four principles enshrined in the 1972 constitution: nationalism, secularism, socialism, and democracy.

**Front Sides (**Fig. 2)**:** The four stars on the Bangladeshi national emblem, along with rice sheaves, tea leaves, and a water lily, stand in for the four tenets of the 1972 constitution [7].

**Fig. 3:**
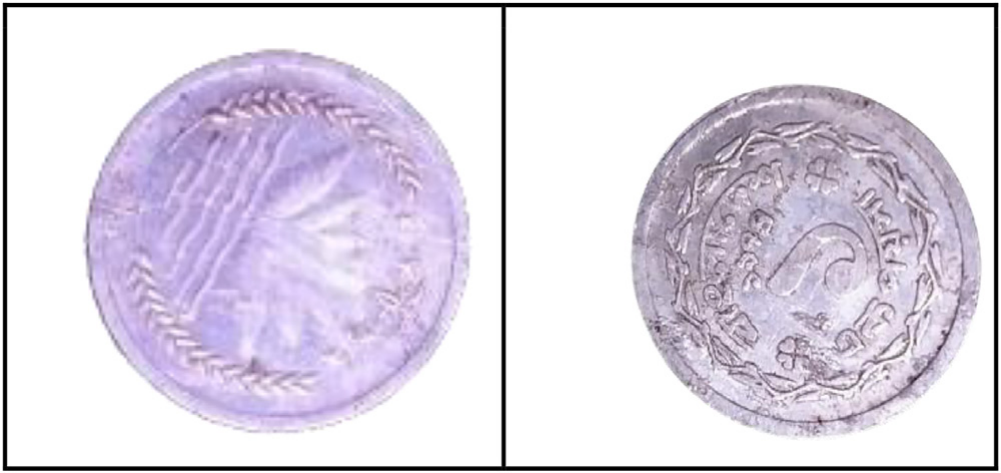
Image of 1 Poisha coin front and backside.

**Back sides** (Fig. 3): The text is a translation of "Bangladesh" from 1 Poisha (1974) with the letters ``বাংলাদেশ'' in Bengali script [7].

### 3.2 Five poisha coin

**Fig. 4:**
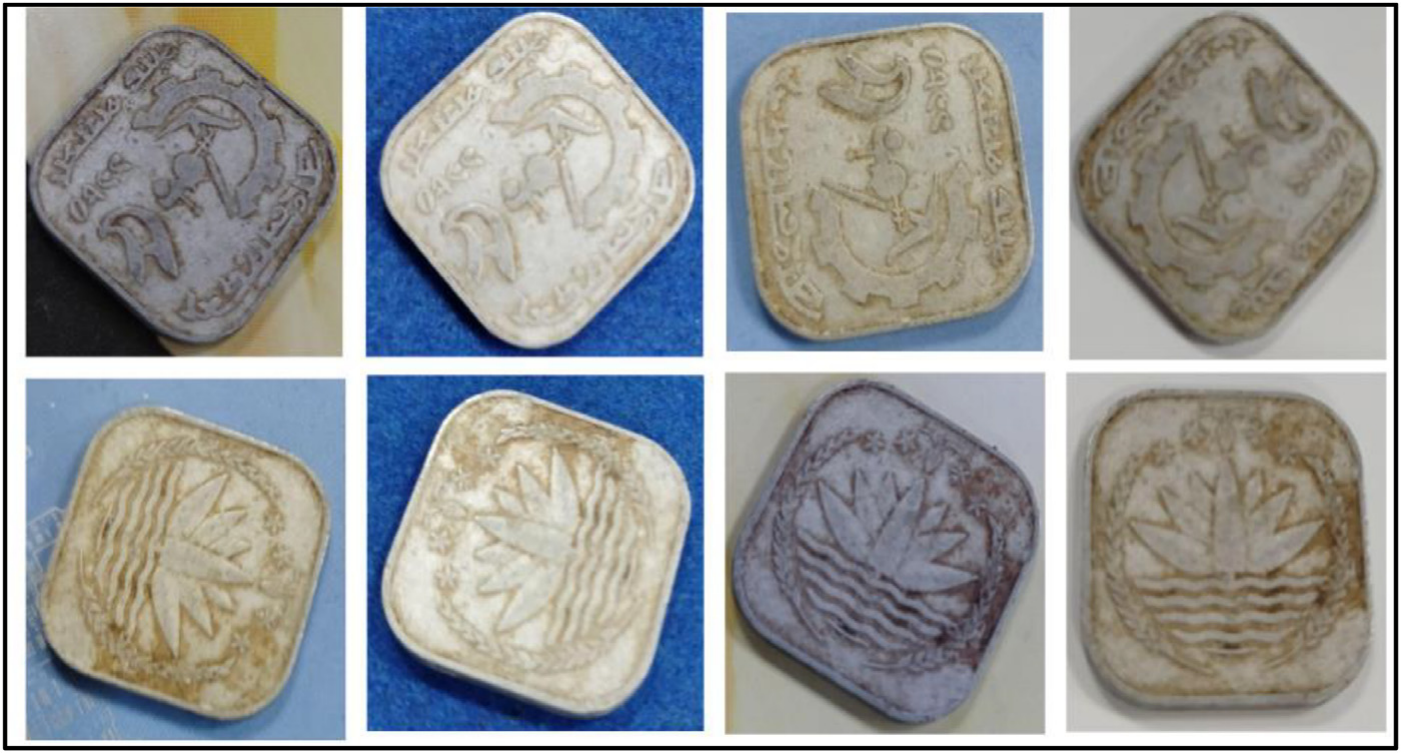
Images of 5 Poisha coin by the years.

**Front Sides** (Fig. 4): A water lily, rice sheaves, tea leaves, and four stars—representing the four tenets of the 1972 constitution—are included in the National Emblem of Bangladesh [7].

**Fig. 5:**
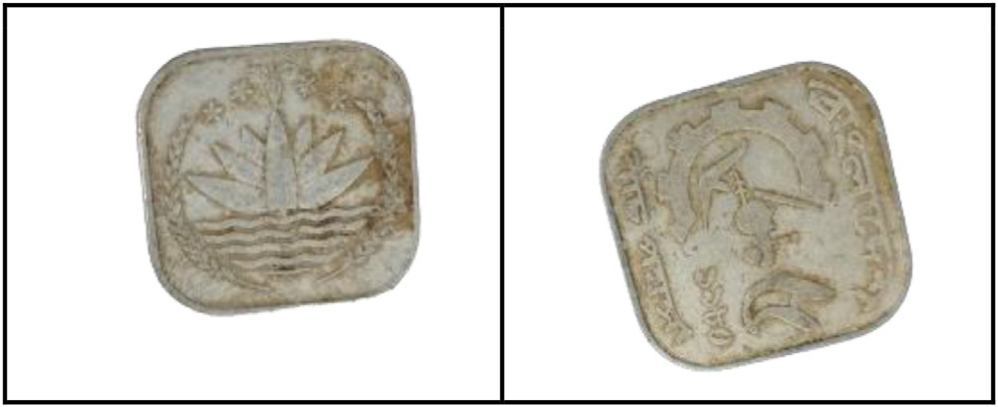
Image of 5 Poisha coin front and backside.

**Back sides** (Fig. 5): A tractor, a swing plough, and a plow are just a few of the agricultural implements, equipment, and transportation techniques that are discussed in the text, along with their country's history and age of use. The text is a translation of "Bangladesh" from 5 Poisha (1980) with the letters ``বাংলাদেশ'' in Bengali script [7].

### 3.3 Ten poisha coin

**Fig. 6:**
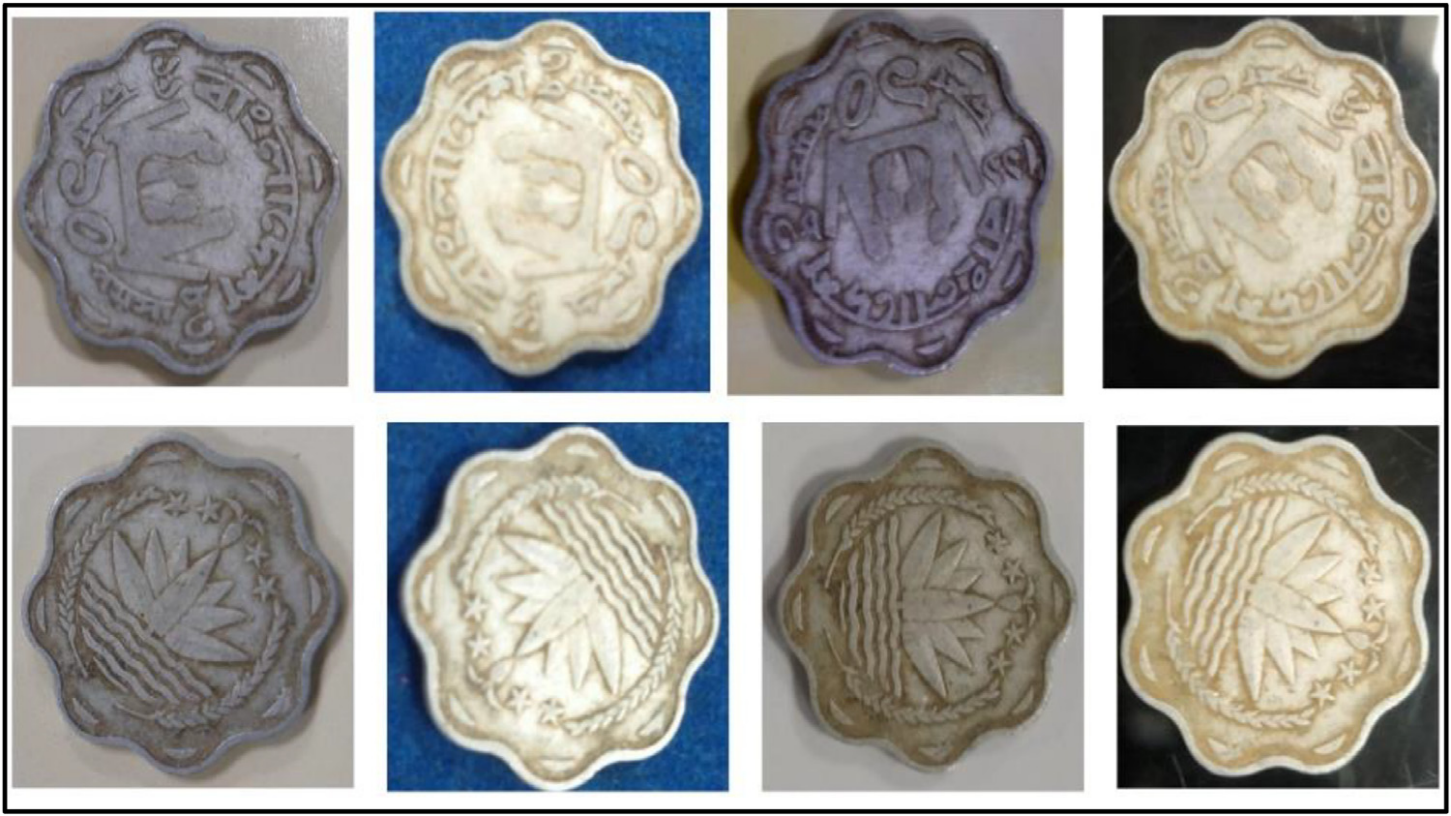
Images of 10 poisha coin by the years.

**Front Sides** (Fig. 6): The national emblem of Bangladesh which was adopted in 1971, features a water lily, rice sheaves, four stars, and three jute leaves. The stars stand for the tenets of nationalism, secularism, socialism, and democracy. The water lily represents Bangladesh's waterways. Rice represents the country's primary food and agriculture [7].

**Fig. 7:**
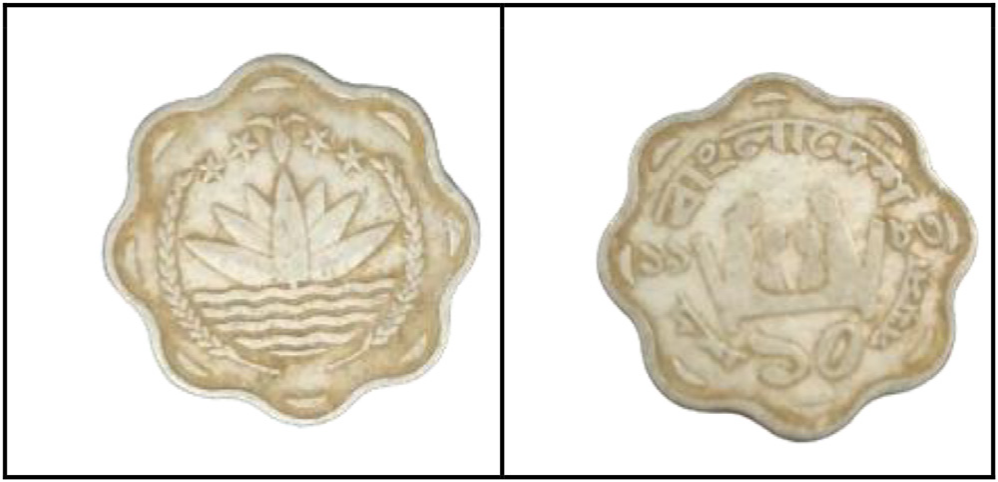
Image of 10 Poisha coin front and backside.

**Back sides** (Fig. 7): A two-child family sitting next to each other, writing the name of the nation and the value of their faces. The text is a translation of "Bangladesh" from 10 Poisha (1983) with the letters ``বাংলাদেশ'' in Bengali script [7].

### 3.4 Twenty-five poisha coin

**Fig. 8:**
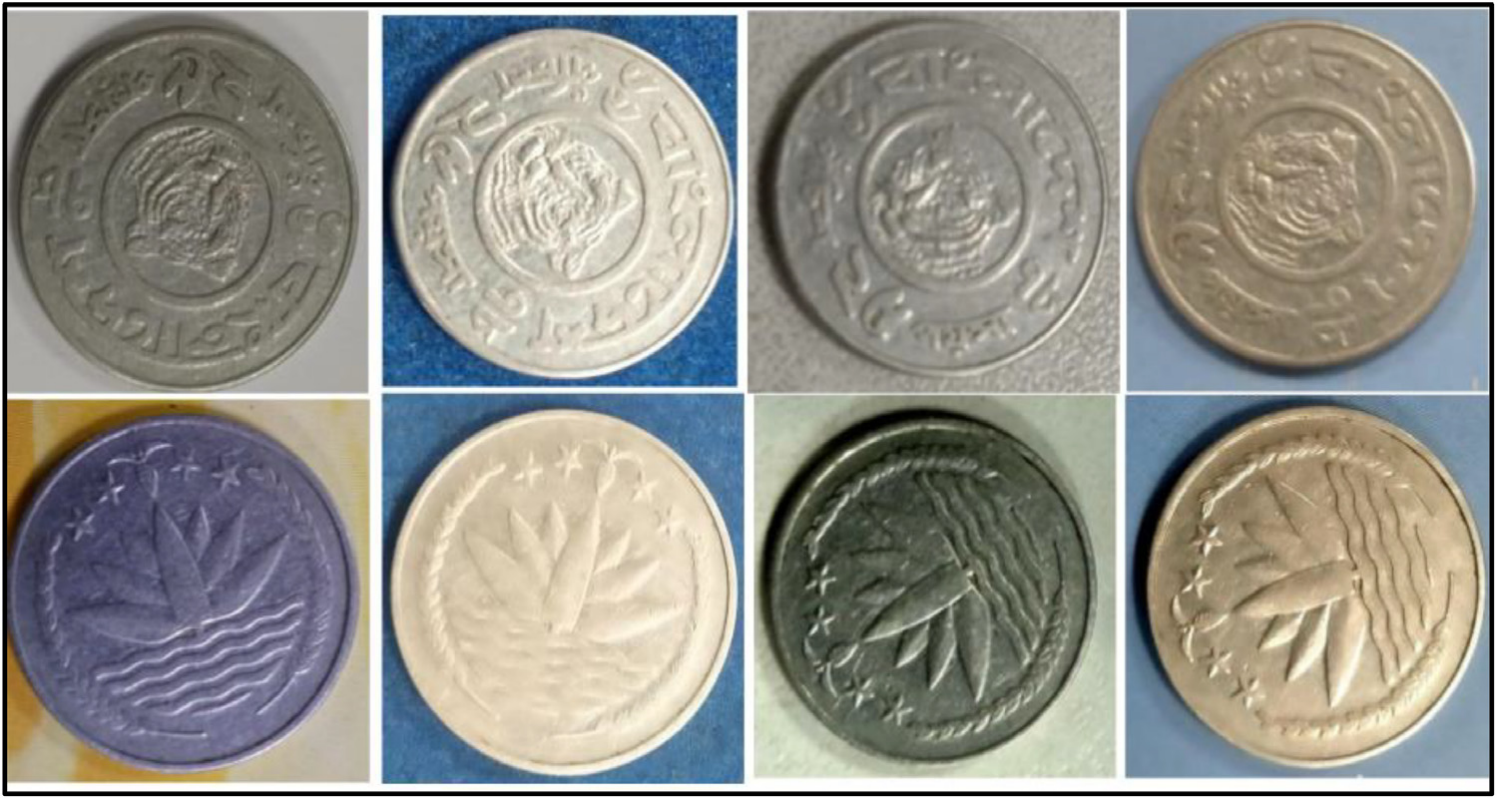
Images of 25 Poisha coin by the years.

**Front Sides** (Fig. 8): The national emblem of Bangladesh which was adopted in 1971, features a water lily, rice sheaves, four stars, and three jute leaves. The water lily symbolizes the country's rivers, rice represents its staple food and agriculture, and the stars represent the founding principles of nationalism, secularism, socialism, and democracy, as enshrined in the 1972 constitution [7].

**Fig. 9:**
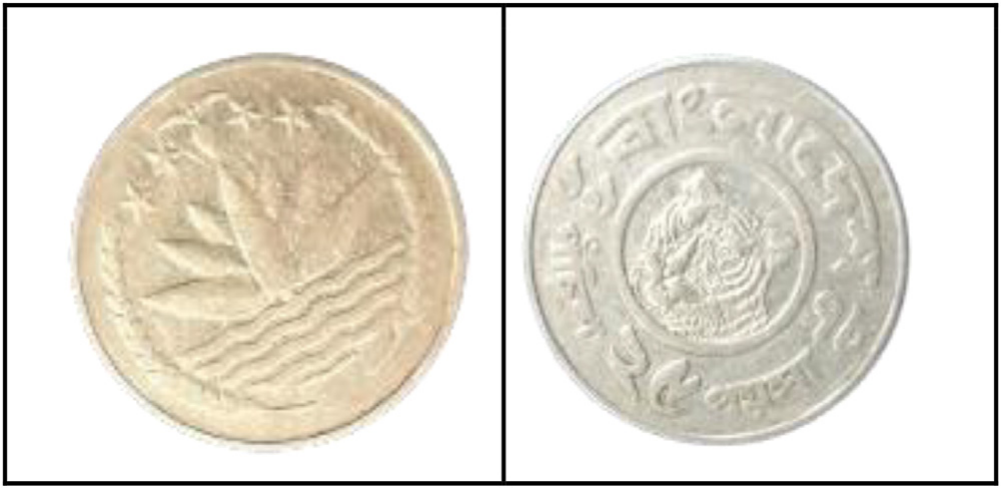
Image of 25 Poisha coin front and backside.

**Back sides** (Fig. 9): The most common subspecies is the Bengal tiger, which is also the national animal of Bangladesh and India. Its coat is yellow to pale orange in color, with dark brown to black stripes. The text is a translation of "Bangladesh" from 25 Poisha (1978) with the letters ``বাংলাদেশ'' in Bengali script [7].

### 3.5 Fifty poisha coin

**Fig. 10:**
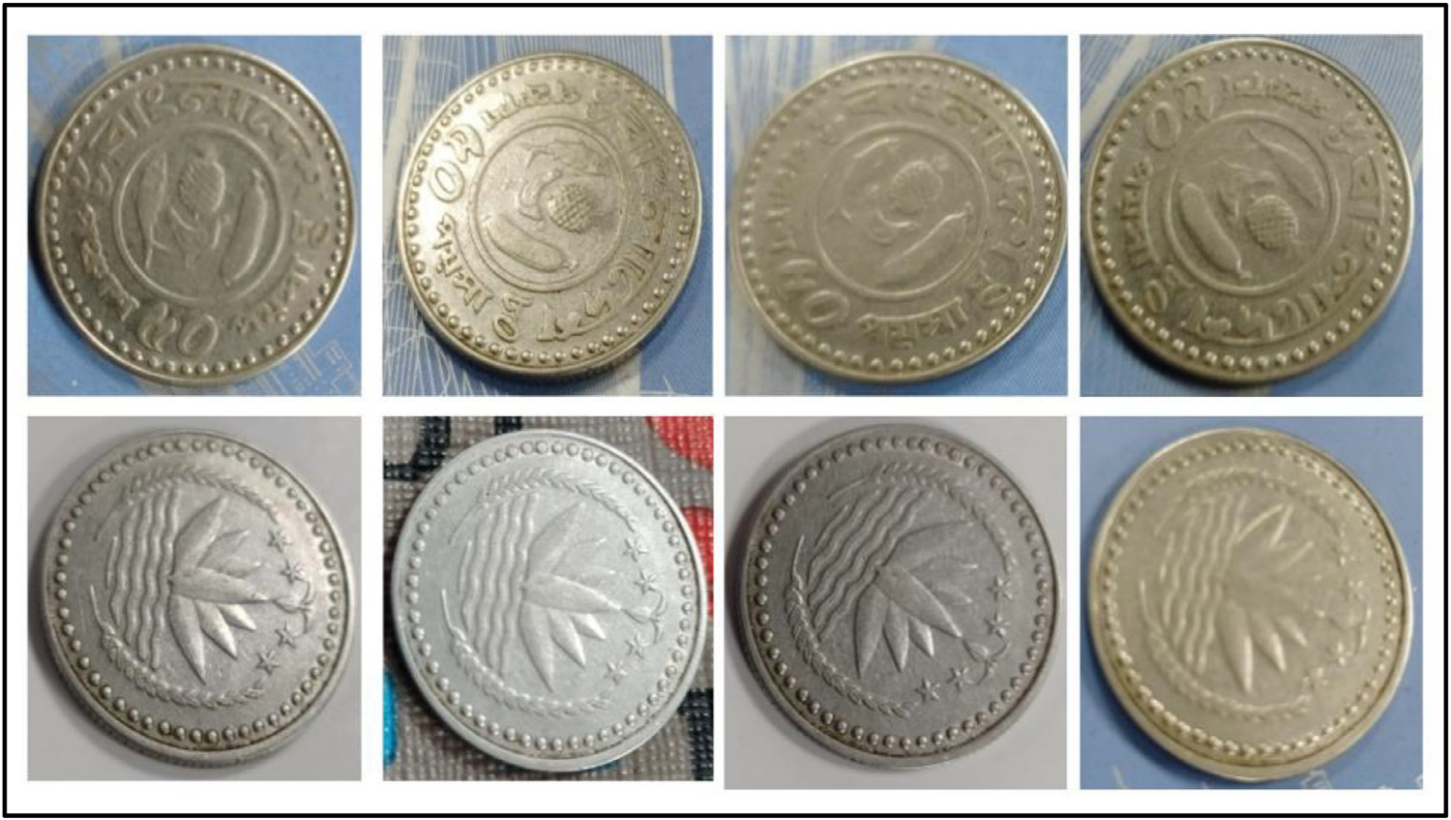
Images of 1 Poisha coin by the years.

**Front Sides** (Fig. 10): Water Lily is Bangladesh's national emblem with rice sheaves on two sides. A three-leaf clover of tea leaves and four stars reflect the four ideals of Bangladesh's first constitution, which was adopted in 1972: nationalism, secularism, socialism, and democracy, respectively [7].

**Fig. 11:**
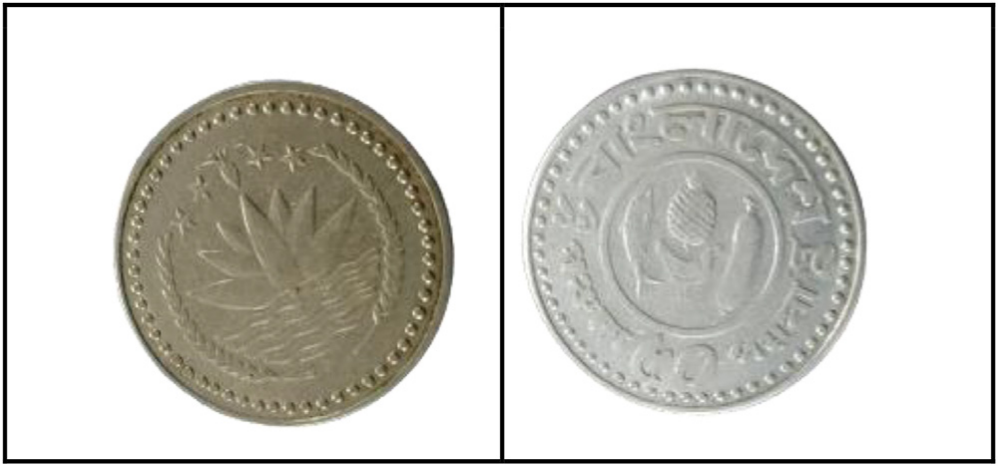
Image of 50 Poisha coin front and backside.

**Back sides** (Fig. 11) : A banana, a pineapple, a chicken, and the national fish of Bangladesh, the hilsa. The text is a translation of "Bangladesh" from 50 Poisha (1980) with the letters ``বাংলাদেশ'' in Bengali script [7].

### 3.6 One taka coin

**Fig. 12:**
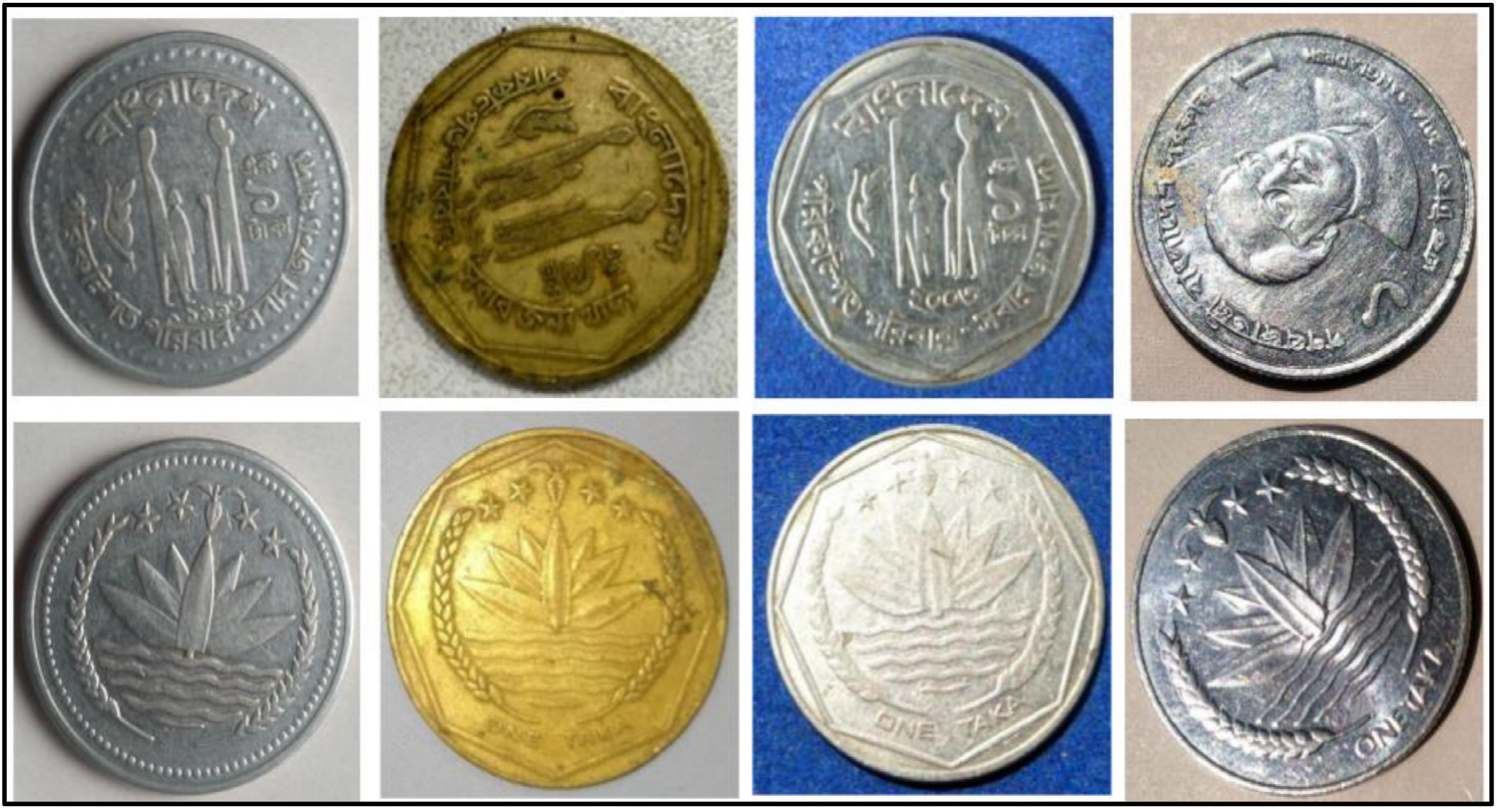
Front and back side images of 1 TakaCoin by the years.

**Front Sides** (Fig. 12): The water lily with two rice sheaves on either side is the national emblem of Bangladesh. The four stars and tea leaves are above [7].

**Fig. 13:**
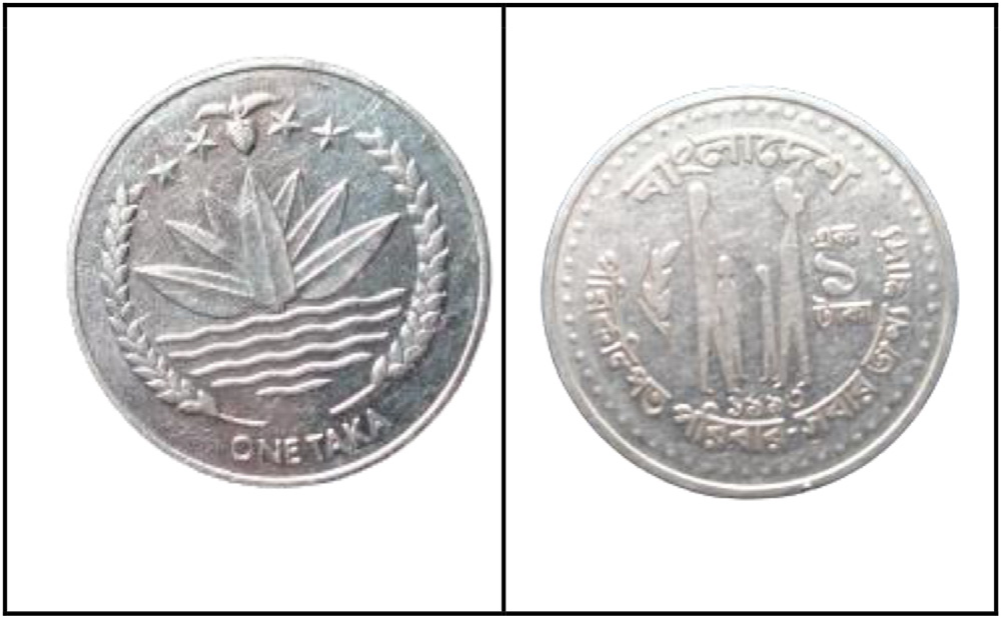
Image of 1 Taka coin front and backside (1993).

**Back sides** (Fig. 13) : Four stylized human figures (parents, son, and daughter) with the headline "Planned Family- Food for All". The text is a translation of "Bangladesh" from 1 taka (1993) with the letters ``বাংলাদেশ'' in Bengali script [7].

### 3.7 One taka coin (1999)

**Fig. 14:**
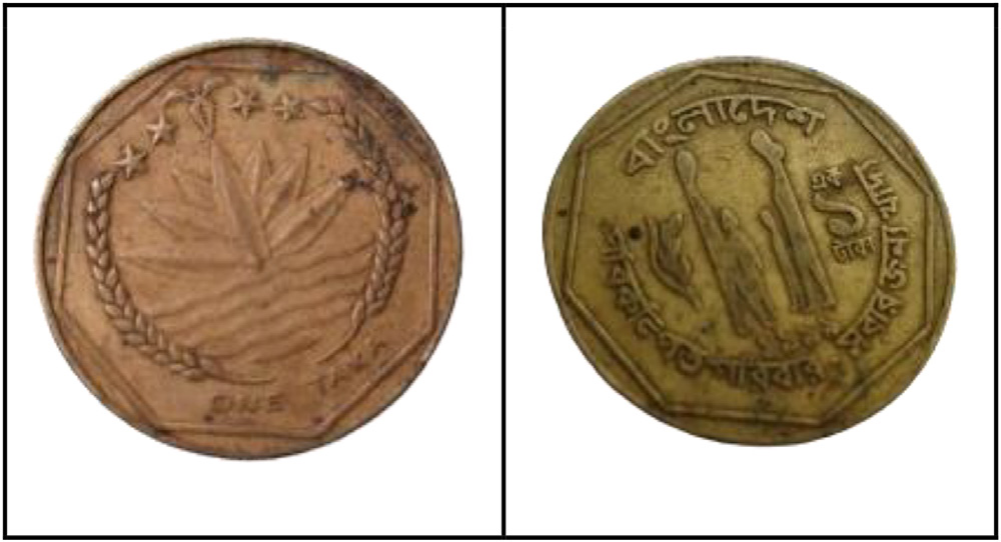
Image of 1 Taka coin front and backside (1999).

**Front Sides** (Fig. 14): The National Emblem of Bangladesh features a water lily, rice ears, three tea leaves, and four stars, symbolizing the four principles of the 1972 constitution [7].

**Back sides** (Fig. 14): Four stylized human figures, including parents with sons and daughters, are displayed with the slogan "Planned Family - Food for All". The text is a translation of "Bangladesh" from 1 taka (1999) with the letters ``বাংলাদেশ'' in Bengali script [7].

### 3.8 One taka coin (2003)

**Fig. 15:**
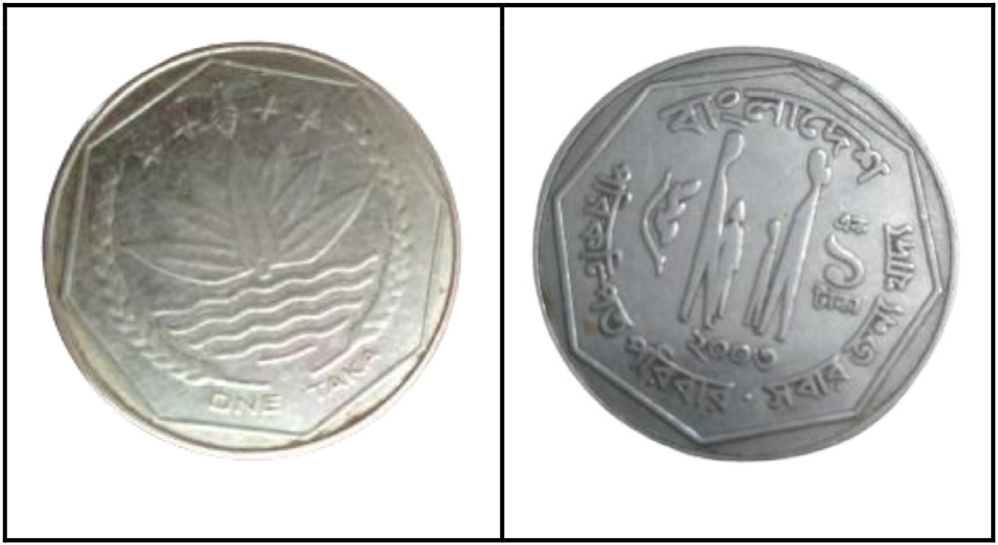
Image of 1 Taka coin front and backside (2003).

**Front Sides** (Fig. 15): The National Emblem of Bangladesh features a water lily, rice ears, tea leaves, and four stars representing the four principles of the 1972 constitution [7].

**Back sides** (Fig. 15): Four stylized human figures, including parents with sons and daughters, are displayed with the slogan "Planned Family - Food for All". The text is a translation of "Bangladesh" from 1 taka (2003) with the letters ``বাংলাদেশ'' in Bengali script [7].

### 3.9 One taka coin (2013)

**Fig. 16:**
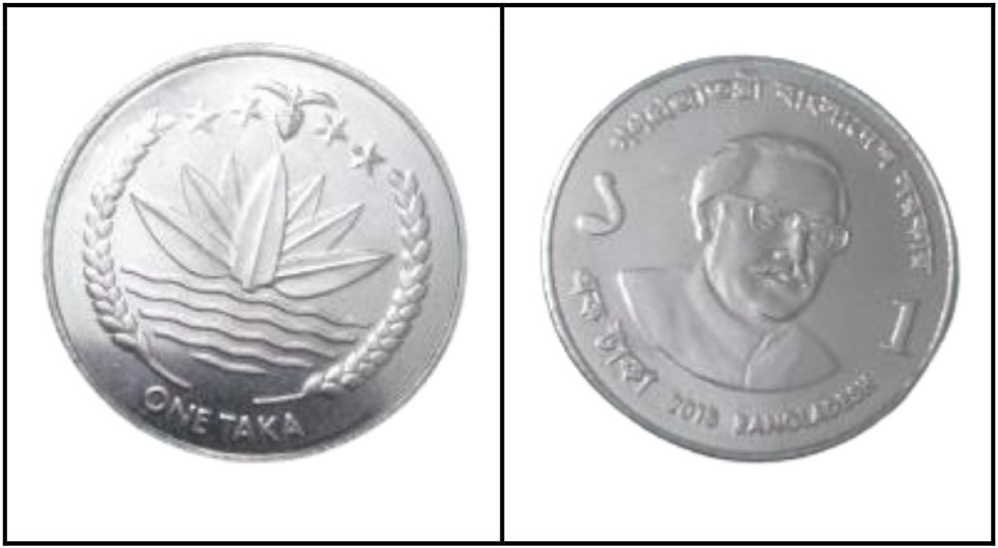
Image of 1 Taka coin front and backside (2013).

**Front Sides** (Fig. 16): Bangladesh's national emblem depicts a water lily, rice ears, tea leaves, and four stars, which represent the four principles of the 1972 constitution [7].

**Back sides** (Fig. 16)**:** The coin features a Bengali issuing authority, a portrait of Sheikh Mujibur Rahman, Bengali alphabet value, Arabic numeral, year of issue, and country name in Bengali and English [7].

### 3.10 Two taka coin (2008)

**Fig. 17:**
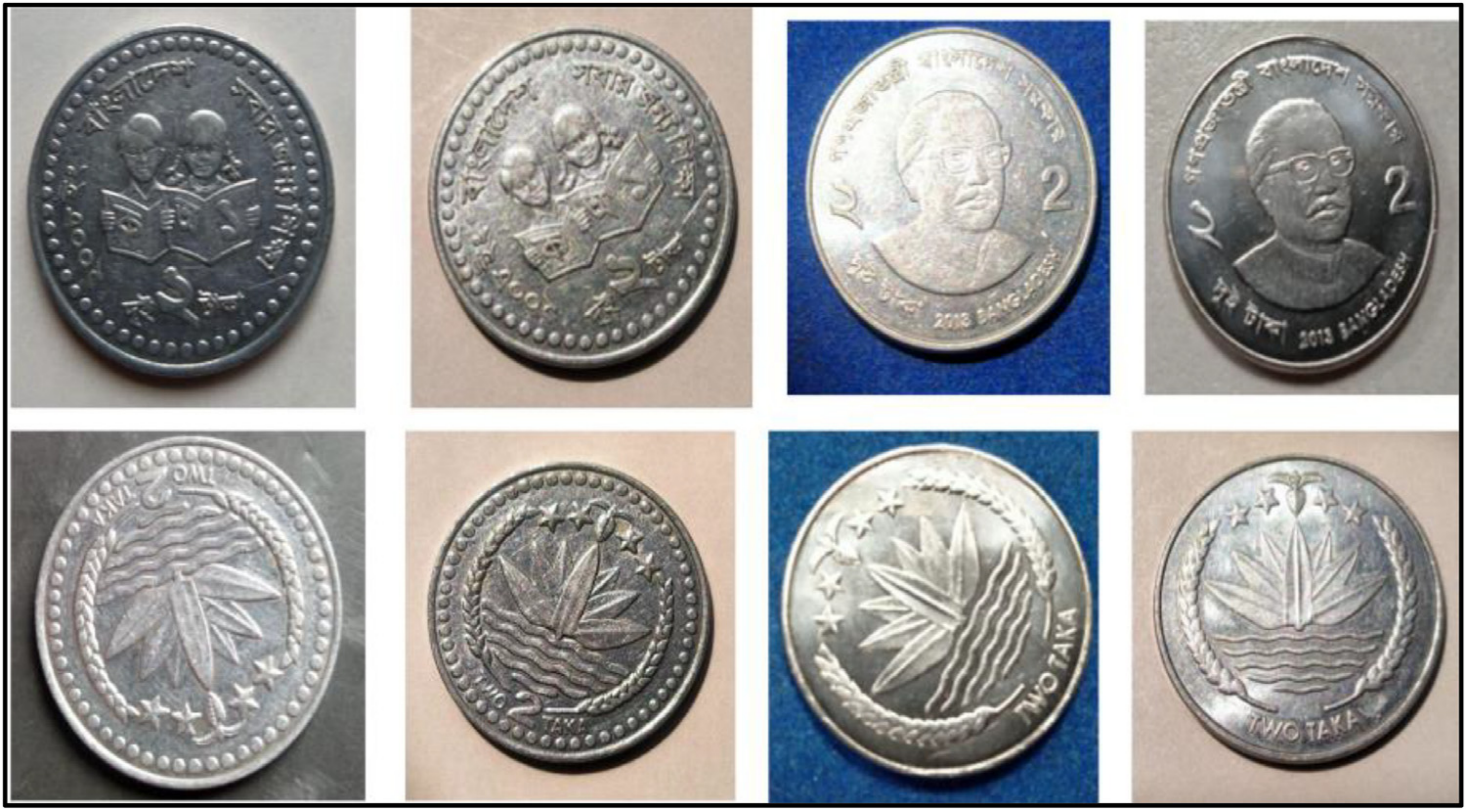
Images of 2 Taka coin by the years.

**Front Sides** (Fig. 17)**:** The face value is below the Bangladesh National Emblem, which is a water lily surrounded by rice sheaves on both sides. A three-leaf clover of tea leaves and four stars illustrate the four principles of Bangladesh's first constitution, established in 1972: nationalism, secularism, socialism, and democracy, respectively [7].

**Fig. 18:**
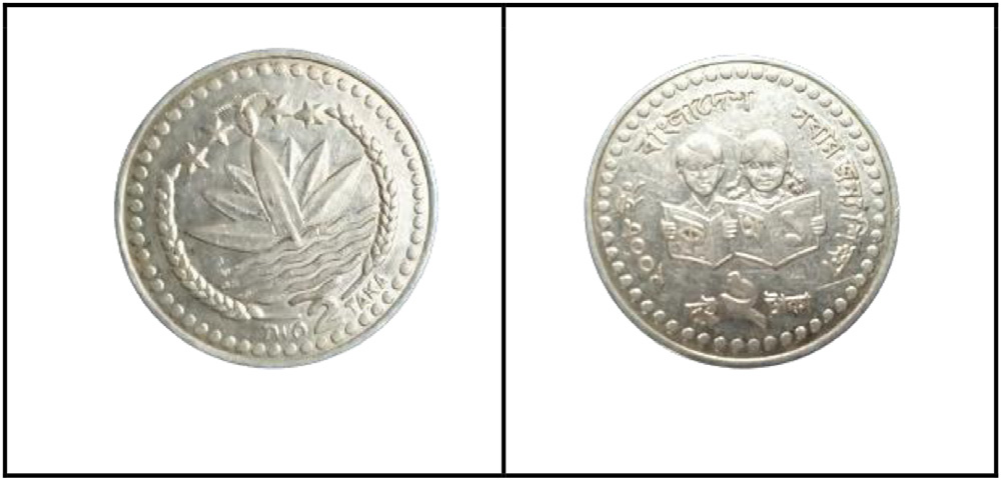
Image of 2 Taka coin front and backside (2008).

**Back sides** (Fig. 18): In the center, a girl and a boy are reading a book. The letters and numerals in the book represent the first alphabets (Bangla has two sets of alphabets) and the first numbers. Around is the year of issuance with the abbreviation ইং GY for Gregorian Year, the name of the country, and the motto on education ``২০০৮ ইং বাংলাদেশ সবার জন্য শিক্ষা''. The face value is shown in figures and in full in the lower section [7].

### 3.11 Two taka coin (2013)

**Front Sides** (Fig. 17): The face value is below the Bangladesh National Emblem, which is a water lily surrounded by rice sheaves on both sides. A three-leaf clover of tea leaves and four stars illustrate the four principles of Bangladesh's first constitution, established in 1972: nationalism, secularism, socialism, and democracy, respectively [7].

**Fig. 19:**
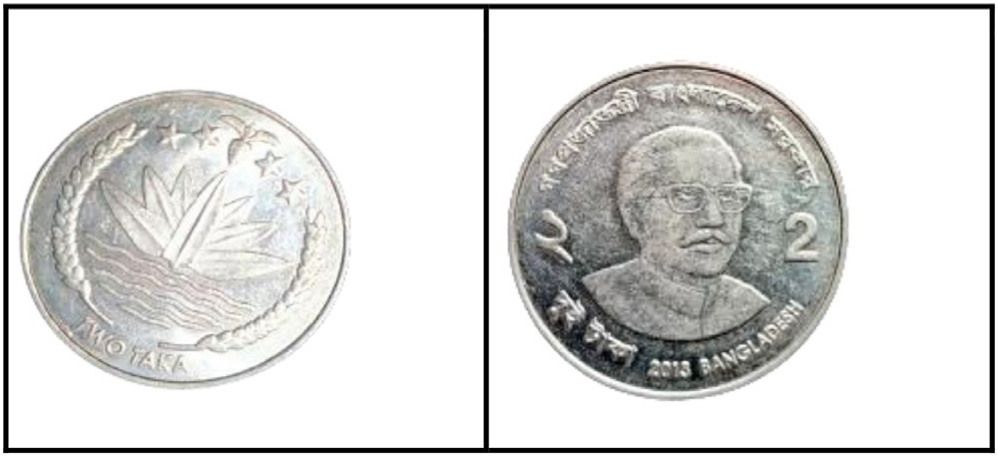
Image of 2 Taka coin front and backside (2013).

**Back sides** (Fig. 19) : The top name of the issuing authority in Bangla is ``গণপ্রজাতন্ত্রী বাংলাদেশ সরকার''. With Sheikh Mujibur Rahman’s portrait in the center, the Father of the Nation. The coin's numerical value is printed on both sides in Bangla and English as ``দুই টাকা 2013 BANGLADESH". The bottom half contains the value in words, the year of issue, and the name of the country [7].

### 3.12 Five taka coin (2008)

**Fig. 20:**
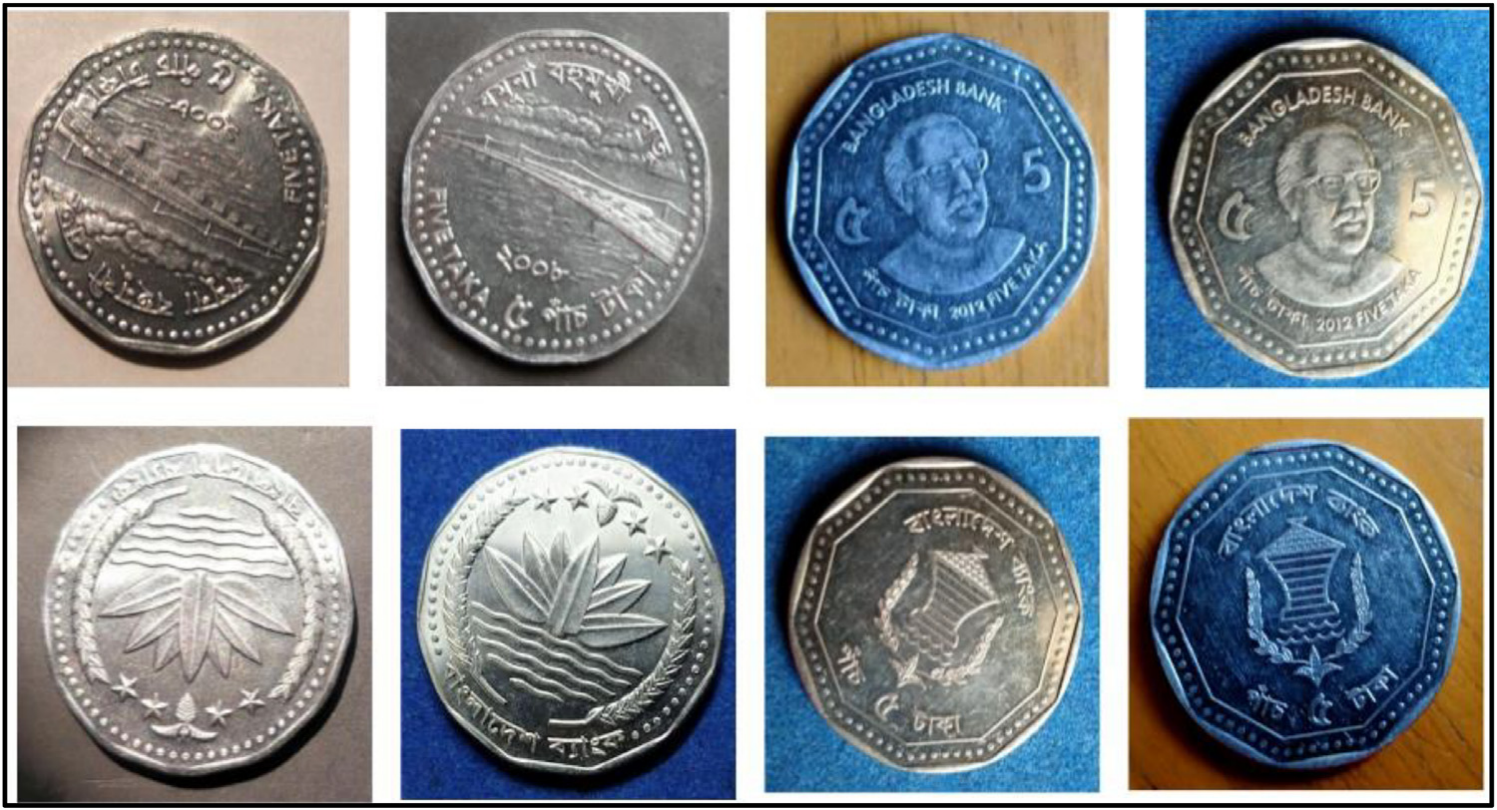
Images of 5 Taka coin by the years.

**Front Sides** (Fig. 20): The National Emblem of Bangladesh features a Water Lily, rice sheaves, tea leaves, and four stars representing the four principles of the 1972 constitution [7].

**Fig. 21:**
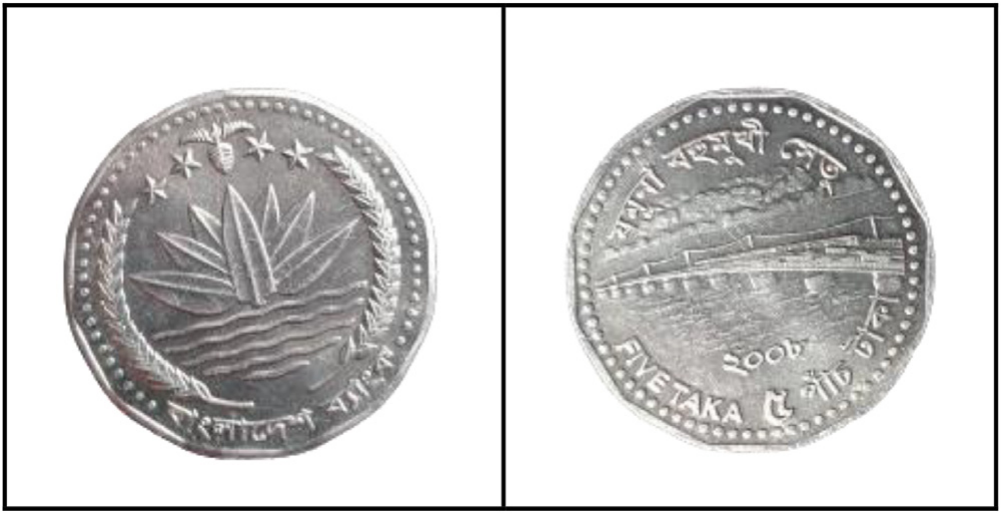
Image of 5 Taka coin front and backside (2008).

**Back sides** (Fig. 21): The top name of the Jamuna Multipurpose Bridge, currently known as Bangabandhu Bridge, is written in Bangla, while the middle shows a photograph of the 5.63-km-long bridge across the Padma River, which is the sixth-longest bridge in South Asia. The text is a translation of "Jamuna Multipurpose Bridge" and "Five Taka 5 Five Taka" from 5 taka (2008) with the letters ``যমুনা বহুমুখী সেতু'' and ``FIVE TAKA ৫ পাঁচ টাকা'' in Bengali script [7].

### 3.13 Five taka coin (2012)

**Fig. 22:**
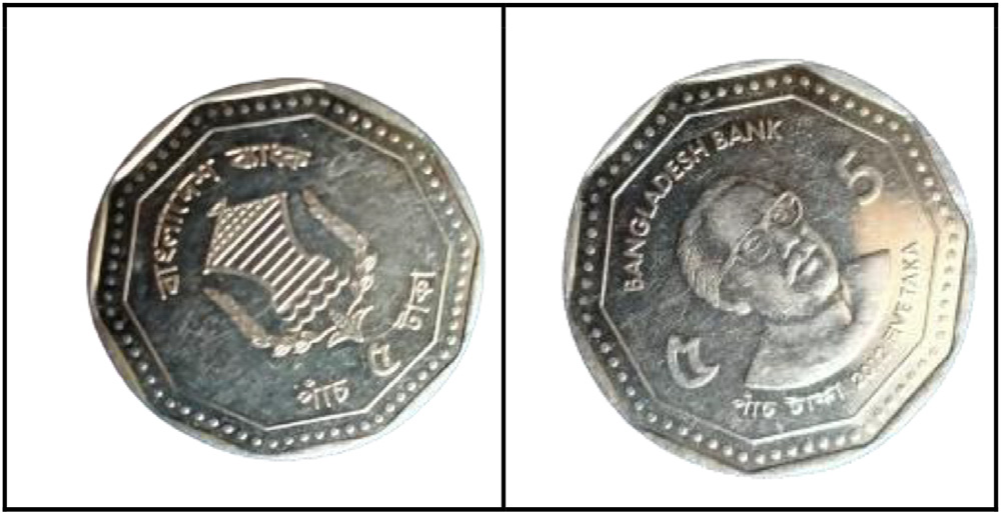
Image of 5 Taka coin front and backside (2012).

**Front Sides** (Fig. 22): March 4, 1972 saw the issuance of the first five notes. Five Taka coins bearing the Bangabandhu Bridge and the national symbol of Bangladesh—a lotus flower, rice grains, jute leaves, stars, and the words "Bangladesh Bank"—were introduced on October 1, 1993 [13].

**Back Sides** (Fig. 22): The top name of the issuing body is "BANGLADESH BANK." The picture of Sheikh Mujibur Rahman, the father of the nation, is in the center. The coin's numerical value is inscribed in Bengali and English on both sides. The text is a translation of "2012 FIVE TAKA" from 5 taka (2012) with the letters ``পাঁচ টাকা'' 2012 FIVE TAKA" in Bengali script [7].

As outlined in Table 2, the dataset provides a detailed breakdown of the number of images for each denomination of Bangladeshi coins. Each class of coins, ranging from 1 Poisha to 5 Taka, has been categorized based on the front and back sides, with the number of images specified for each category. The dataset is further organized into folders by coin denomination, providing a well-structured collection for analysis.

**Table 2:** Full description of coin dataset.

Name of the coins	Class	Category	Number of Images	Folder Name
1 Poisha Coin	1	Back	345	1Paisa_1974Back
	2	Front	386	1Paisa_1974Front
5 Poisha Coin	1	Back	416	5Paisa_1980Back
	2	Front	451	5Paisa_1980Front
10 Poisha Coin	1	Back	422	10Paisa_1983Back
	2	Front	459	10Paisa_1983Front
25 Poisha Coin	1	Back	416	25Paisa_1978Back
	2	Front	421	25Paisa_1978Front
50 Poisha Coin	1	Back	469	50Paisa_1980Back
	2	Front	479	50Paisa_1980Front
1 Taka Coin	1	Back	356	1Taka_1993Back
	2	Front	356	1Taka_1993Front
	3	Back	350	1Taka_1999Back
	4	Front	340	1Taka_1999Front
	5	Back	353	1Taka_2003Back
	6	Front	340	1Taka_2003Front
	7	Back	336	1Taka_2014Back
	8	Front	396	1Taka_2014Front
2 Taka Coin	1	Back	378	2Taka_2008Back
	2	Front	315	2Taka_2008Front
	3	Back	325	2Taka_2013Back
	4	Front	351	2Taka_2013Front
5 Taka Coin	1	Back	358	5Taka_2008Back
	2	Front	379	5Taka_2008Front
	3	Back	413	5Taka_2012Back
	4	Front	373	5Taka_2012Front
**Total**	**26 Classes**		**9983 Images**	26 Folders

## 4 Experimental design, materials and methods

### 4.1 Dataset collection and preparation

The dataset collection process was designed to ensure consistency, reproducibility, and comprehensive coverage of each coin category. The initial impetus came from a 1 Poisha coin—minted in 1973 and retrieved from a family collection—that sparked interest in documenting Bangladesh’s coinage heritage. This personal connection laid the groundwork for a systematic and methodical approach to data acquisition.

**Fig. 23:**
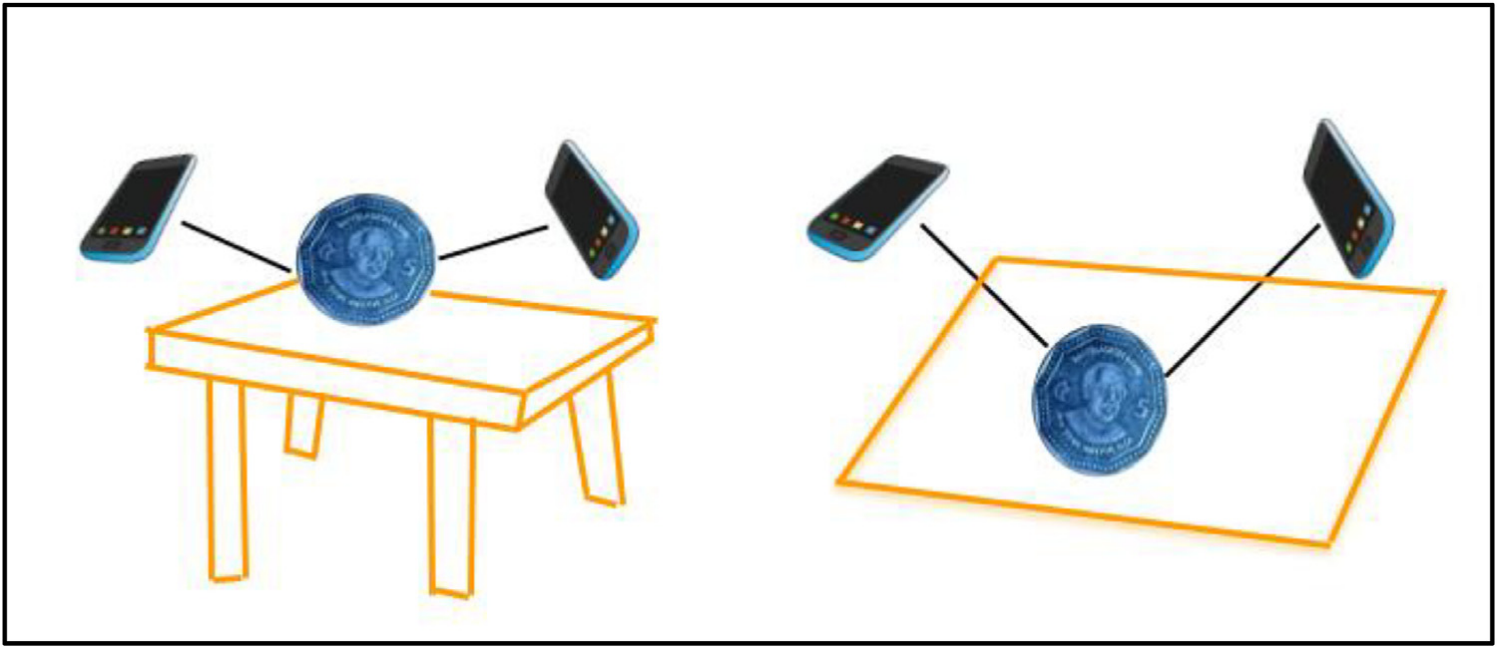
Dataset collection.

To achieve a representative collection, coins were sourced from multiple avenues: vintage specimens from the streets of Gulistan, Dhaka—a renowned marketplace for collectors—ensured inclusion of older denominations (1 Poisha, 5 Poisha, 10 Poisha, 25 Poisha, and 50 Poisha), while more recent issues (1 Taka, 2 Taka, and 5 Taka) were obtained from everyday household currency reserves. A visit to the Taka Museum in Dhaka further enriched the dataset, introducing rare and commemorative coins, unique mint marks, and specimens illustrating minting anomalies. This multi-sourced approach provided a diverse range of coins that reflect both the historical and contemporary landscape of Bangladeshi coinage. The specific camera resolutions and configurations are provided in Table 3. Fig. 23 illustrates the steps taken during this process, from color adjustments to image resizing, highlighting the methods used to enhance the dataset's quality.

**Table 3:** Device information of capturing the images.

Device Name	Camera Resolution
Realme 5S	Quad camera with 48 MP f/1.8 wide angle main camera, 1/2.0″, 0.8 µm, PDAF, 8 MP f/2.2 13 mm ultrawide lens, 2 MP f/2.4 macro lens and 2 MP f/2.4 depth camera with LED flash, HDR, panorama modes.
Oppo A5 2020	Quad camera with 12 MP f/1.8 26 mm wide angle main camera, 1/2.8″, 1.25 µm, PDAF, 8 MP, 119˚ ultrawide lens, 1/4.0″, 1.12 µm, 2 MP, f/2.4, depth camera and 2 MP, f/2.4 depth lens. Including the LED flash, HDR, panorama modes

Each coin was meticulously prepared prior to photography, ensuring that surfaces were free of dust and that reflective glare was minimized. To maintain consistency, the following standardized procedure was adopted for image capture:

1. **Uniform Background and Setup**: All coins were placed against a neutral, non-reflective backdrop (e.g., a matte white foam board) to eliminate background distractions and simplify segmentation. Care was taken to ensure that the surface was flat, stable, and evenly lit.
2. **Controlled Lighting Conditions**: Artificial light sources with diffusers were employed to maintain uniform illumination and reduce harsh shadows. The lighting intensity and angle were kept consistent throughout the imaging sessions, and where possible, natural light was minimized to ensure reproducible lighting conditions. In cases where ambient conditions varied (e.g., due to weather or time of day), adjustments to exposure and camera settings were made to maintain a consistent visual quality.
3. **Camera Setup and Angles**: Two smartphones—Realme 5S and Oppo A5 2020—were utilized for imaging due to their high-resolution sensors and versatile lens configurations (see Table 3). To capture both obverse and reverse sides, each coin was photographed from a top-down angle, ensuring the camera’s lens remained perpendicular to the coin’s surface to avoid perspective distortion. Coins were then rotated or tilted slightly to capture multiple views, documenting details such as minting defects, lettering, and edge patterns.
4. **Resolution and Image Quality Controls**: Each image was taken at the highest resolution supported by the device’s primary camera to preserve fine details. Multiple shots were taken for each coin to select the clearest, most uniformly lit image. Images were initially stored in their native format before subsequent processing steps (e.g., resizing, color adjustments) were applied uniformly to the entire dataset.
5. **Metadata and Cataloging**: For each coin, metadata such as denomination, year of minting (if legible), source location, and any notable characteristics (e.g., commemorative issue, mint error) were recorded in a structured log. This ensured traceability and allowed for subsequent analysis correlating image features with historical and numismatic attributes.

### Dataset Preparation

To further enhance the dataset, we applied a range of geometric modifications and advanced methods during the dataset preparation process:

• **Color Space Transformation**: We randomly adjusted the brightness, contrast, and RGB color channels of the images. This step ensures that the model is robust to variations in color and illumination, which are common in real-world scenarios.
• **Resizing Images**: Each image was resized to 700 by 700 pixels. This resizing not only reduces computational complexity but also retains crucial information for accurate analysis, allowing the model to better learn from and analyze the images while building overall accuracy across the dataset.

## Limitations

While this study presents a comprehensive dataset of Bangladeshi coins and highlights its potential applications in computer vision, machine learning, and accessibility technologies, there are several limitations that should be considered:

1. **Limited Geographic and Temporal Scope**: The dataset primarily focuses on coins from Bangladesh, and while it includes various denominations, it may not represent the full diversity of global coinage or capture all historical variants of Bangladeshi coins. Coins from other regions or time periods could have added further value to the dataset.
2. **Environmental and Lighting Conditions**: Although we applied techniques to simulate varying lighting conditions through color space transformation, the dataset does not encompass all possible real-world environments. The model may still face challenges when exposed to extreme variations in lighting, weather conditions, or backgrounds not present in the dataset.
3. **Resolution Constraints**: The resizing of images to 700×700 pixels was necessary for computational efficiency, but this could result in a loss of finer details that may be important for certain tasks, such as detecting subtle features or minting errors on coins.

## Ethics statement

The authors adhere to the journal's ethical guidelines and confirm that this research does not involve humans, animals, or data obtained from social media. The datasets utilized in the study are publicly accessible, and appropriate citation protocols should be followed when utilizing these datasets.

## CRediT author statement

**Mahamudul Hasan:** Conceptualization, Methodology, Supervision, Visualization, Project administration, Validation; **Krittika Roy:** Supervision, Investigation, Methodology, Supervision, Writing – original draft, Writing – review & editing; **Nowshin Tasnia:** Supervision, Investigation, Methodology, Supervision, Writing – original draft, Writing – review & editing; **Dr. Mohammad Rifat Ahmmad Rashid:** Conceptualization, Methodology, Supervision, Visualization, Project administration, Validation.

## Data Availability

Mendeley DataNumismatic Heritage of Coin Dataset Bangladesh (Original data)
